# Parental Perceptions of Child’s Play in the Post-Digital Era: Parents’ Dilemma with Digital Formats Informing the Kindergarten Curriculum

**DOI:** 10.3390/children10010101

**Published:** 2023-01-03

**Authors:** Andreja Istenič, Violeta Rosanda, Marina Volk, Mateja Gačnik

**Affiliations:** 1 Faculty of Education, University of Primorska, Cankarjeva 5, 6000 Koper, Slovenia; 2 Institute of Psychology and Education, Kazan Federal University, Kremlyovskaya 18, 420000 Kazan, Russia

**Keywords:** digital toys, digital learning technology, digital technology, parents, infants, early learning, kindergarten, play, post-digital era

## Abstract

Digital technology affordance has been recognized as a social and learning tool, and the requirements for digitalizing the kindergarten curriculum have been present for decades. Digitalization in a child’s early years can present conflict with parents, as the societal and economic demands of digitalized society for a child’s digital technology use are in dissonance with guidelines and recommendations of health organizations that caution against preschool children’s technology use. Kindergarten curricular reform in Slovenia was conducted in the 1990s. In this period, the use of digital learning technology and digital play for the development of children learning predispositions and early literacy was already recognized. At the time of curriculum design, it integrated some elements of digital learning technology. Now, 30 years after the design of the new curriculum, we are facing the post-digital era. Learning technology in the early years is a matter of partnership with parents; accordingly, this descriptive survey study includes a non-randomized sample of 306 parents. We are considering how child’s play is structured in the primary environment and how parents perceive digital technology in the current post-digital age of seamless, digitally saturated social practices. Parents are aware of the risks of technology and of its benefits for learning. The findings show correlations between a child’s digital screen technology use and parents’ attitudes and perceptions of digital play. Parents that offer digital screen technology to a child have less agreeable attitudes regarding its possible risks to a child.

## 1. Introduction

The requirements for digitalizing the kindergarten curriculum have been present for decades. Slovenia’s play-based learning kindergarten curriculum [1] is the result of the kindergarten curricular reform conducted during the last decade of the 20th century. The main objective was the optimal condition for child development, learning and emerging abilities. This curriculum is based on sociocultural theory originating from Vygotsky, which recognized the child’s primary cultural environment within a family and the child’s interactions within. Kindergarten’s role in enabling a child’s learning by utilizing all play genres, such as social and symbolic play utilizing ZPD, for all children, also those from home environments offering fewer incentives and providing for them a continuum in development, learning and emerging abilities. The recognition of the role of interactions emerged with experience, and as such, the parent’s role in a child’s primary environment and its play is under consideration in the post-digital era. The use of digital learning technology in social interactions and digital play is considered for the development of children learning predispositions and early literacy.

Digitalization requirements had changed at the time of curriculum reform at the end of the 20th century. The diversity and differences in the kindergarten class were noted as one of the main teacher’s challenges and the use of ICT for optimizing learning in terms of individualized instruction [2], early literacy [3,4,5], developing mathematical concepts [6] and learning about ICT and its manipulation [7]. Plowman et al. [7] categorized digital technology in learning as (1) learning about digital technology and how to manipulate it, (2) expanding knowledge about the world in curricular areas and (3) developing affective, social and cognitive dispositions for learning. A child’s authentic activity is play, and learning is conducted by play; therefore, a kindergarten teacher needs to be informed by the child’s play engagements in their home environment. The presented study examined how parents construct preschoolers’ home environment and play in terms of a share of digital play vs. traditional, how they perceive digital technology and digital play and its role in a child’s development and learning vs. traditional play.

### 1.1. Digital Technology in Play-Based Learning Kindergarten Curriculum: The Role of Partnership with Parents and Interaction between Primary and Secondary Environment

Diverse factors drive the need for change and reform in the kindergarten curriculum. Economic development and 21st-century skills [8] are based on outcome orientation and measure effectiveness and efficiency according to learning outcomes in elementary schools, resulting in employability. The motivation behind the education and industry, and government and nongovernment authorities promoting digital technology for digital skills and STEM professions to secure future workforce are in dissonance with recommendations of public health agencies advocating for reduced technology use [9].

Early learning researchers focus on a child’s authentic activity and its role in development and learning. They recognize the realities of children’s home environments and the growing role of digital technology in preschoolers’ lives [10,11]. Sociocultural theories originate in Vygotsky [12], which highlights the role of a child’s cultural environment and the communication and interaction of a child with a more experienced person, in which artefacts and tools play an important role. Digital technology has been recognized as an important social and learning tool that influences social practices [13]. The recognition of the role of interaction has emerged with more experience, and as such, the parent’s role in a child’s primary environment is again under consideration in the post-digital era, with digital technology transforming parent–child interactions.

Kindergarten curriculum reform in Slovenia at the end of the 20th century. The main objective was the optimal condition for child learning and developing a child’s emerging abilities. A play-based learning kindergarten curriculum was introduced involving diverse areas, including movement, language, nature, society, art and mathematics. The reform considered the transition away from a predominantly care-based institution, introducing an inclusive learning environment for all children, and enabling an equalizing, process-based curriculum in early literacy [1]. The main theory-driven curricular reform was the sociocultural theory based on Vygotsky established the base for curricular activities that also support children from home environments in which incentives are not high [14]. The critical element was language in early literacy promotion, which addressed the zone of proximal development in the context of the play, enabling graphomotor skills, metalinguistic awareness and storytelling, and symbolic play for ages five and a half to six [14].

The use of digital learning technology for the development of children’s learning predispositions and early literacy was recognized at the time of curricular reform at the end of the 20th century. The diversity and differences in the kindergarten class were noted as one of the main challenges to teachers and the use of ICT for optimizing learning in terms of individualized instruction [2]. The use of digital technology in kindergarten for early literacy was discussed in two ways: how technology supports early literacy and how ICT changes it [3]. Cook [3] discussed its use in exploratory play and symbolic representations by applying writing and drawing and advocating for ICT, offering a greater range of creative possibilities than traditional graphic and print tools.

Plowman et al. [7] categorized digital technology for learning by the following: (1) learning about digital technology and how to manipulate it; (2) expanding knowledge about the world in curricular areas; and (3) developing affective, social and cognitive dispositions for learning. A child’s activity is play, and a child’s learning takes place during play. Therefore, a kindergarten teacher needs to be informed by child’s play engagements in their home environment. Play with digital technology involves all play genres as a child uses artefacts and tools in epistemic, functional and ludic play [15,16].

Analyzing the integration in curriculum identified were activities for the threefold model according to Plowman et al. [17]. The Slovene kindergarten curriculum [1] involves activities for (1) understanding digital media and manipulation abilities, for example: manipulating digital devices (camera, voice recorder, taking photos); (2) expanding understanding of the world in various curricular subjects (learning games and educational programs, reading and storytelling, creative engagement with drawing, scripts, comic strips, etc.); (3) developing dispositions for learning in the affective, social, cognitive areas (dance, drama, video performance, listening and imitating sounds, audio-visual activities, etc.). The first set of criticism refers to the integration of digital technology isolated to learn about technology and develop skills, and the second set of criticism refers to the lack of integration of digital technology across subject areas [18], which allows for authentic learning and application of technology.

In the Slovene curriculum, the recommendations are not on the level of curriculum objectives as in some other countries. For example, the Australian children’s education and care quality authority [19] includes digital technology within the learning objective of “the communicatively successful child” within the preschool curriculum [19]. The standard description consists of the child working with different texts and developing meaning (multimedia texts are mentioned in this connection), the child presenting ideas and establishing meaning using different media, and the child using ICT to access information, explore ideas and present their thinking [19]. The Slovene guidelines [20] for the use of digital technology according to the curriculum [1] mention digital technology in line with the principles of equity, diversity and multiculturalism, the principle of privacy and intimacy, the principle of partnership with parents, the principle of cooperation with the community, the principle of active learning enabling verbalization and other modes of expression.

Partnership with parents is among basic principles, as parents’ perceptions of a child’s learning and development influence a child in their primary environment and connection to the secondary environment in kindergarten. The development of emergent literacy takes place in day-to-day interactions in the family environment, utilizing a set of objects and artefacts, including digital technology [21]. Integrating digital technology can be difficult for parents, as the balance between its benefits and harms requires informed decisions. Therefore, educators’ evidence-based interventions are needed to support parents of young children [22]. Studies report parents’ raising concerns about digital technology use in kindergartens [23], which also needs to be addressed by educators. Addressing the contexts in which digital technology is used in the early period requires the involvement of parents as active stakeholders who mediate the child’s activities [21].

### 1.2. Guidelines for Digital Technology by Health Associations

In 2021, the Medical Chamber of Slovenia published a strict set of screen viewing recommendations [24] for a child until the age of two, stating they should not be exposed to screens at all. From two to five years of age, the time limit should be less than one hour a day, increasing in proportion to age and only in the presence of parents (roughly 20 min a day for a three-year-old child or 30 min a day for a four-year-old child). Only between the ages of six and nine can a child look at the screen longer but should not spend more than one hour a day in front of the screen. [24]. The guidelines by health organizations and relevant bodies are adapting to the changes that digitalization brings to society. The American Academy of Pediatrics recommends incorporating activities with touch interfaces starting at 18 months of age. For the age period between the second and fourth years, they recommend the use of high-quality learning technology to be accompanied by the interaction of parents with toddlers [25]. The Canadian Paediatric Society puts the benefits for learning and development first, followed by the risks [26]. Australian health organization guidelines advise against viewing screens and using digital technology before age two. Recommendations also concern parent–child interactions and the limited involvement of digital media [27].

### 1.3. Parents’ Attitudes between Benefits and Uncertainty of Digital Technology in Child’s Learning and Development

How parents understand the parent–child relationship and the role of toys and objects and digital technology are essential in parents’ integration of digital technology in a home environment. Parents’ perceptions and attitudes of the technology’s role in a child’s life are essential when a child’s play environment is mediated by parents [28,29,30]. If we want to examine a child’s experiences, it is necessary to understand parents’ perceptions [31]. In a child’s primary environment, parents’ digital technology use sets the ground for a child’s digital behavior. The selection and provision of digital technology and digital toys are in the parents’ domain [10]. The perception of parental involvement in child play refers to interaction, guidance, and screen engagement [32,33].

Parental awareness involving digital technology is growing; however, due to increasing information on its benefits and harm, parents constantly need information and guidelines. In addition, age levels are decreasing in terms of the reach of media exposure and a variety of experiences that parents need to be made aware of. The reported dissonance between public health guidelines reducing screen time and educational perspectives on digital technology [9] creates tension and uncertainty among parents [23].

Several studies report parents’ conflicting views of digital technology in a child’s life and, among them, positive influence on a child’s literacy learning and development and awareness of the negative effect on child development [23,34,35,36]. Studies reported parents’ concerns about digital technology use in kindergarten [23]. Parents’ attitudes toward digital play are examined in related studies. The study across four countries, China, South Korea, the US and Turkey, reported that digital toys and digital play were the least preferred play across all four countries [37]. A South Korean study reported increasing societal and economic tension that digital play brings into homes, changing parental practices for preparing a child for competitive education and the job market [38]. The research reports tablet use among preschool children [10,11] daily [39] for a set of activities like viewing a video, viewing family photos, digital storytelling [28,40], music creation [41], and art and drawing skills [42]. Applications are becoming widespread in early childhood [43,44,45]. In a study of 124 apps advertised as educational, a large share of 72 (58%) were considered of lower quality [46], p. 10.

### 1.4. Post-Digital Materiality of Child’s Primary Environment

A child’s learning and development take place when a child manipulates and control the approximate environment. The cognitive theory highlights the physical and mental actions engaging materiality [47,48]. Physicality and connection of the body and the mind are inseparable in a child’s cognitive processes, and physical and cognitive development go hand in hand P [47]. Sociocultural theories emphasize the role of social interaction and the interaction with/by artefacts. Bruner [48] designed a threefold model identifying representations that children utilize in learning, including the enactive, visual and symbolic. Verbal and non-verbal interaction between a mother and a child and later between a child and other people is essential in a child’s learning and development. Sensory experience in environment exploration is primary.

In the post-digital era, the environment is constructed in societal processes utilizing physical and digital materiality. If, in the digital era, the focus of pedagogical research and practice was digital technology and its functions, the post-digital era focuses on the transition made to examine social practices that seamlessly integrate digital technology and, as such co-constitute social practices [29,49,50]. The proliferation of digital technologies is affecting interaction and socialization as the perception of reality (materiality of physical and digital and transmedia practices) and the child’s agency [28]. In the post-digital era, materiality needs to be reexamined, establishing the interaction process in the environment formed by digital technology. For the earliest period, the interaction of parents with the child is important, in which non-verbal communication and eye contact play an important role. Marsh discussed the role of the relationship between material and discursive [51] and new forms of multimodal meaning-making [21]. Emergent literacy develops on the boundary of online and offline integrating digital technology and engaging in the involved multimodal texts [21]. We were interested in how parents perceive the inclusion of traditional and electronic toys and their role in parent–child communication. We examined how parents explain a child’s behavior and interaction with different means, and the effects of a child’s interaction with electronic versus traditional means.

Digital technology has been recognized as an important social tool that has proliferated in diverse sociocultural practices. Among cultural artefacts, digit artefacts expand their share, and their influence on social practices is increasing. Empirical research recognizes the importance of digital tools in a child’s cultural environment [10,11], and the communication modes and social interactions are determined by digital tools [13]. A child’s use of digital tools as social tools and as learning technology enables them to learn about the culture.

Digital artefacts are present within a child’s play; as indicated by Marsh [21], the boundaries between analogue and digital are blurred. In today’s society, early literacy emerges in a post-digital context, indicating that digital is significant to literacies. Therefore, Marsh et al. [21] define literacy as digital literacy, as it no longer concerns only writing and takes place by applying a variety of digital technologies in social practices reflecting online and offline activities.

Research reports that digital technology enters children’s play at all ages [17,30,39]. Along with traditional toys, children encounter screen-based digital toys and non-screen digital toys [30] and are espoused to touch screens and a range of digital media from birth [17]. A child utilizes a range of devices and tools in creative play, and it is necessary to explore what forms a child’s play. Parents select and offer toys and artefacts to a child. Researchers need to examine parents’ perceptions. In some countries, digital technology is not integrated into the kindergarten curriculum, and it is necessary to bridge the gap between the consumption of entertaining popular culture and learning technology [52].

We classify toys [36] into four categories and add digital screen technology and non-digital objects and artefacts:

- Traditional toys (toys not running on batteries, electricity or solar power);
- Simple electric and electronic toys without screens (battery-powered, electric or solar-powered, but not involving computer technology);
- Digital non-screen toys (toys with computer technology but without a screen; some can be connected to the Internet);
- Digital screen toys (toys with computer technology and a screen that run on batteries, electricity or solar power);
- Digital screen technology with screens that is not primarily for gaming but can also be used for gaming (smartphone and tablet, laptop, PC, etc.);
- Non-digital objects and artefacts;

Research questions:

- RQ1: How do parents perceive digital technology and its role in a child’s development and learning?
- RQ2: How do parents perceive characteristics of child’s play with traditional and digital toys?
- RQ3: How do parents construct preschoolers’ home environment and play regarding a share of digital play vs. traditional?

## 2. Materials and Methods

### 2.1. Research Design and Instruments

An online survey was conducted among parents of preschoolers. The descriptive study examines the attitudes and perceptions of parents about play and how parents structure a child’s play by offering a set of toys.

The online questionnaire was designed based on a review of studies [31,39,44,45,53,54,55,56,57,58,59]. The first part consisted of descriptive questions about a child (gender, age, position in a family regarding being the first, second or single child), descriptive questions about a parent (parent role, gender, age), and living environment (urban, suburb, rural). The second part of the questionnaire consisted of questions about play and toys, apps and the child’s daily activities. The third part considered parents’ attitudes about digital play’s contribution to learning and development and their perception of child’s play. The fourth part considered communication during the play, skills development and the child’s reactions when playing with toys. The fourth part is not presented in this paper.

The instrument scale measuring parents’ attitudes consist of items from a scale by Vittrup et al. [31] and Wooldridge [32]. The items from Vittrup et al. [31] include that media exposure at a young age (0–3 years) is important for early brain development; A child will not fall behind other children academically if his or her use of technology tools is restricted in the early years (0–3 years); Children under the age of 2 years should have no TV screen time; The use of a computer can promote long-term physical, emotional, or intellectual developmental damage; Introducing technological tools at a young age prepares children better for tomorrow’s workforce.

Based on Wooldridge [32], the following items were included: Young children develop better without technology; My child should first learn to interact with the physical world; they have a lifetime to interact with the virtual world; I am concerned about the effect technology can have on a young child’s brain development and learning; I want the child to enjoy their childhood and not become addicted to technology; If my child does not master technology, he or she will be excluded from peer groups; Due to the intensive use of digital technology in the family, parents are reading less and less to their children.

The section on parental perception of child’s play with traditional and digital toys consisted of items from a related study [33]: Children under three should not use technological toys. Some technological toys enhance a child to develop early literacy. Technology toys enhance children to be passive. According to our research questions, we designed additional items that included reflecting child–parent interaction, how parent guide play [48], child preferences between traditional and digital, and digital toys facilitating digital competency.

Daily playtime activities were designed based on the study zero to eight [39] consisting of items describing TV viewing, game playing and integrating traditional outdoor play activities, reading, and listening to music.

The section about types of toys consisted of a classification of toys made by authors [36] based on a review of related studies. Parents were asked to assess the percentage of the amount of playtime in a way that total play time is 100%. They could select from a range of types of toys: traditional toys (toys not running on batteries, electricity or solar power); simple electric and electronic toys without screens (battery-powered, electric or solar-powered, but not involving computer technology); digital non-screen toys (toys with computer technology but without a screen—some can be connected to the Internet); digital screen toys (toys with computer technology and a screen that run on batteries, electricity or solar power); digital screen technology with screens that is not primarily for gaming but can also be used for gaming (smartphone and tablet, laptop, PC, etc.); non-digital objects and artefacts.

The section about applications was designed based on review [39,44,53] and consisted of items describing categories and not focusing on specific characters or games. Items were educational games apps, maker space apps, transmedia character apps, daily habit routines apps, storytelling apps, and entertaining apps.

The reliability of the instrument indicates sufficient Cronbach alpha between 0.79 and 0.92.

### 2.2. Data Collection

Data were collected with an online questionnaire in the spring from March to May 2021. Before starting the questionnaire, parents agreed to written consent regarding anonymity, aggregated data analysis and reporting.

### 2.3. Sample

The non-randomized sample consisted of 277 mothers (90%) and 29 fathers (10%). Parents reported living environment; among them, 165 were from the urban environment, 72 from the suburban area and 69 from the rural area. Parents reported the gender of their child and included 48% of girls, and 52% were boys. The average child’s age in the total sample was 3.6 years of age. The child parents reported in the survey were 119 the youngest child, 17 were middle children, 80 were the oldest children, and 90 were only children.

### 2.4. Data Analysis

Descriptive statistics, means and standard deviations are presented. Computed was the Spearman correlation coefficient, and, based on findings, correlations between age and dependent variables and correlations between digital screen technology and dependent variables were presented. The rule of thumb ρ < 0.19 negligible correlation; ρ = 0.20–0.39 weak correlation; ρ = 0.40–0.59 moderate correlation; ρ = 0.60–0.79 strong correlation; ρ ≥ 0.80 very strong correlation was used.

## 3. Results

Results are presented in the subsections according to the research questions.

### 3.1. How do Parents Perceive Digital Technology and Its Role in Child Development and Learning?

**Figure 1:**
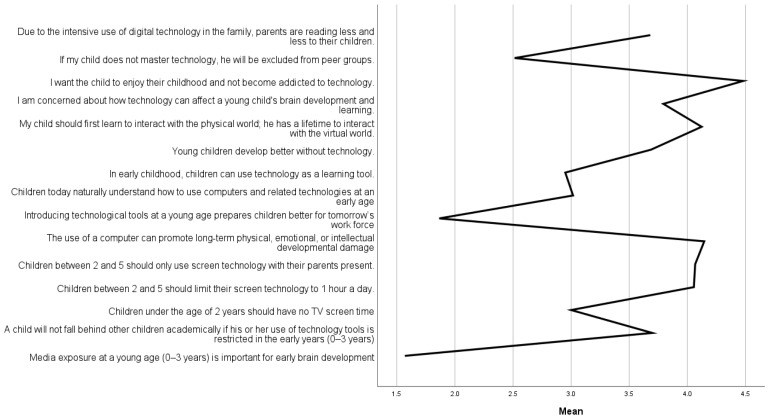
Parents’ attitudes towards digital technology in child’s development and learning.

Parents are aware of the risk, and potential harm digital technology brings to a child’s life (Figure 1). The highest means indicate items describing risks and parents’ attitudes towards limiting and supervising their child’s technology use. The highest mean involved the item I want the child to enjoy their childhood and not become addicted to technology (M = 4.48, SD = 0.73). High means are also for items the use of a computer can promote long-term physical, emotional, or intellectual developmental damage (M = 4.15, SD = 0.90), and my child should first learn to interact with the physical world, he/she has a lifetime to interact with the virtual world (M = 4.12, SD = 0.99). Parents would limit screen time to an hour daily (M = 4.06, SD = 0.99), and they believe that screen technology could only be used under the supervision of parents (M = 4.07, SD = 0.85). Further, parents indicate concern about the influence digital technology has on a child’s development and learning (M = 3.79, SD = 1.01) and believe that a child develops better without digital technology (M = 3.69, SD = 1.06). Parents also believe that a child will not fall behind other children academically if his or her use of technology tools is restricted in the early years (0–3 years) (M = 3.70, SD = 1.37).

**Figure 2:**
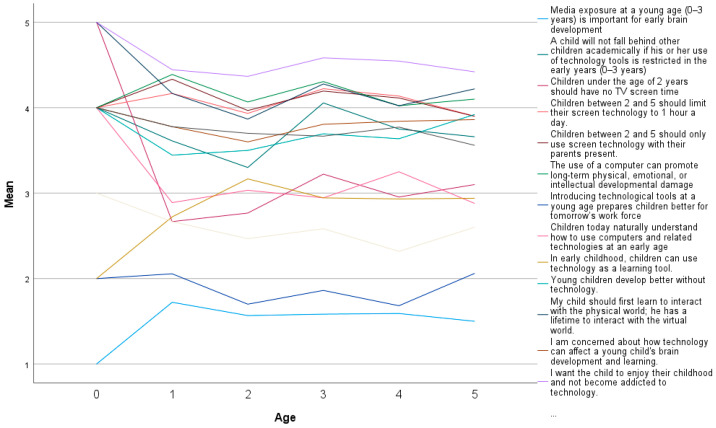
Parents’ attitudes towards digital technology in child’s development and learning according to child’s age.

There are some differences between age groups, as seen in Figure 2. From the age of three, parents hold a stronger view that a child will not fall behind other children academically if his or her use of technology tools is restricted in the early years (0–3 years) (M = 4.06, SD = 1.12). For other items, there were no differences in means between age groups. Between boys and girls, the mean difference is indicated for the item children today naturally understand how to use computers and related technologies at an early age for boys indicating lover mean (M = 3.09, SD = 1.08) vs. girls (M = 3.93, SD = 1.08).

The correlations between the use of digital screen technology with parents’ attitudes are presented in Table 1. Pearson correlation coefficient indicates a negative correlation on a level *p* = 0.01 (two-tailed) with parents’ attitudes about technology harming a child. The highest correlations are for items: the use of a computer can promote long-term physical, emotional, or intellectual developmental damage (ρ = −0.406, *p* < 0.001), a child develops better without technology (ρ = −0.436, *p* < 0.001), I want the child to enjoy their childhood and not become addicted to technology (ρ = −0.421, *p* < 0.001) and I am concerned about the effect technology can have on a young child’s brain development and learning (ρ = −0.384, *p* < 0.001). Strangely parents who offer digital screen technology to a child agree with statements and have positive correlation reported: media exposure at a young age (0–3 years) is important for early brain development (ρ = 0.451, *p* < 0.001), introducing technological tools at a young age prepares children better for tomorrow’s workforce (ρ = 0.476, *p* < 0.001) and in early childhood, children can use technology as a learning tool (ρ = 0.359, *p* < 0.001).

### 3.2. How Do Parents Perceive Characteristics of Child’s Play with Traditional and Digital Toys?

**Figure 3:**
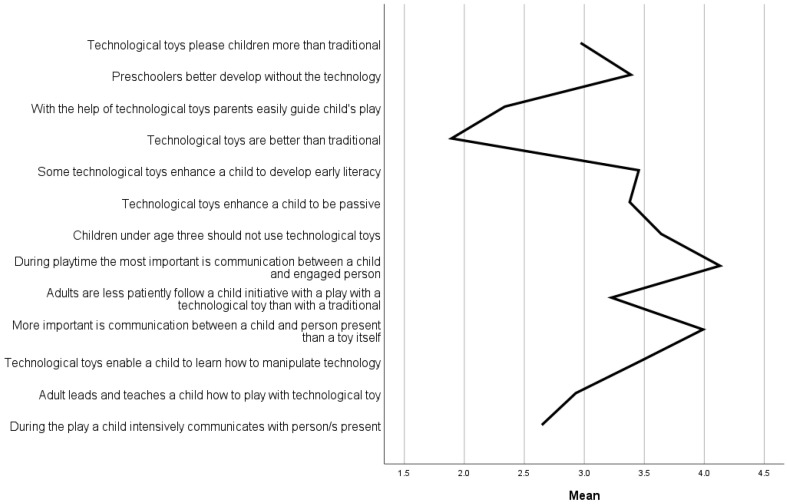
Parents perceive characteristics of child’s play with traditional and digital toys.

Parents’ perceptions of their child’s play are presented in Figure 3. The highest means are for items during the play most important is communication (M = 4.13, SD = 0.84) and more important than a toy is communication (M = 3.99, SD = 0.91) however, communication is not as much observed as present during the play with a technological toy (M = 2.65, SD = 1.2).

Means for negative attitudes about play with digital technology are indicated in statements preschoolers better develop without the technology (M = 3.39, SD = 1.02), with the highest means for 2-year-olds (M = 3.50, SD = 1.26) and 5-year-olds (M = 3.50, SD = 0.97). Technology toys enhance children to be passive (M = 3.38, SD = 1.00) with the highest means for five-year-olds (M = 3.52, SD = 1.00). Children under three should not use technological toys (M = 3.64, SD = 1.06), with the highest mean for five-year-olds (M = 3.93, SD = 0.93).

**Figure 4:**
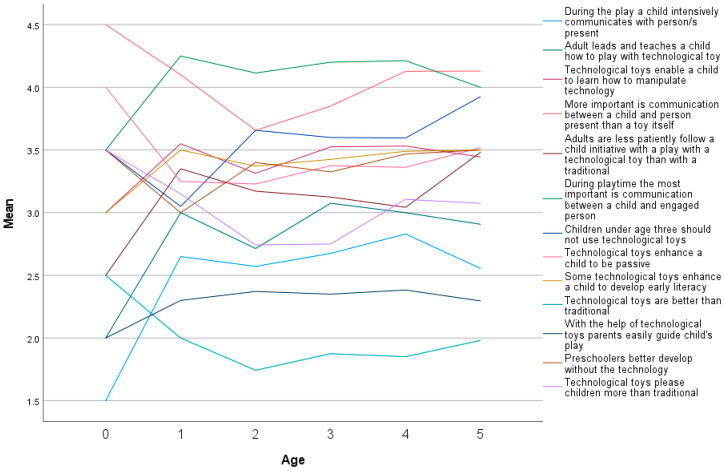
Parents perceive characteristics of a child’s play with traditional and digital toys according to the child’s age.

How parents perceive learning according to age groups is presented in Figure 4. Some technological toys enhance a child to develop early literacy (M = 3.45, SD = 0.82), with the highest mean for five-year-olds (M = 3.50, SD = 0.80) and one-year-olds (M = 3.50, SD = 0.67). The second was the statement technological toys enable a child to learn how to manipulate technology (M = 3.46, SD = 0.84), with the highest means for one-year-olds (M = 3.55, SD = 0.60) and three-year-olds (M = 3.53, SD = 1.01) as also for four-year-olds (M = 3.53, SD = 0.86).

The higher mean difference between genders is for technological toys please children more than traditional, with the girls’ mean higher (M = 3.12, SD = 1.18) vs. boys’ mean (M = 2.84, SD = 1.04). The means are very similar for boys and girls regarding negative parents’ attitudes; children under the age of three should not use technological toys, and technological toys enhance a child to be passive and vary for the item preschoolers better develop without the technology with girls’ mean higher (M = 3.53, SD = 1.09) vs. boys mean (M = 3.26, SD = 0.95).

Regarding educational benefits, there is not much difference between means; more in item technological toys enable a child to learn how to manipulate technology, with girls’ mean higher (M = 3.55, SD = 0.92) vs. boys’ mean (M = 3.39, SD = 0.76), while in item some technological toys enhance a child to develop early literacy with girls’ mean lower (M = 3.40, SD = 0.90) vs. boys’ mean (M = 3.50, SD = 0.73).

The use of digital screen technology indicates a correlation on a level *p* = 0.01 level (two-tailed) with parents’ perceptions of toys (Table 2). The highest correlation is with perceiving technological toys as superior to traditional ones (ρ = 0.405, *p* < 0.001). Observed are negative correlations with preschoolers would better develop without technology (ρ = −0.364, *p* < 0.001) and under the age of three technological toys should not be in use (ρ = −0.284, *p* < 0.001), technological toys enhance a child’s passivity (ρ = −0.203, *p* < 0.004) and also that communication is most important in play (ρ = −0.152, *p* < 0.033).

### 3.3. How Do Parents Construct Preschoolers’ Home Environment and Play in Terms of a Share of Digital Play vs. Traditional?

**Figure 5:**
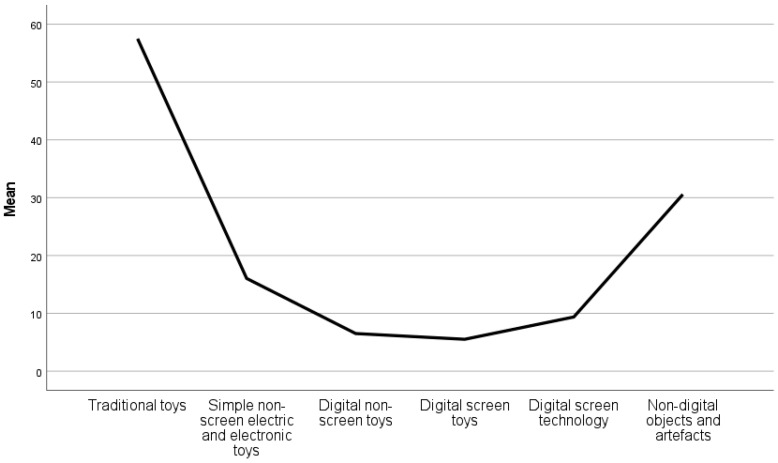
Parents’ estimation of playtime with traditional and digital toys.

In Figure 5, the playtime according to the share of a variety of toys is presented. The largest share constituting child’s play by traditional toys (57.50%), followed by non-digital objects and artefacts (30.57%). In third place are simple non-screen electric and electronic toys (16.05%). Digital technology constitutes together almost a fourth of all play time (21.42%). Digital technology among this is the largest share (9.38%), including digital non-screen toys (6.52%) and digital screen toys (5.52%).

The use of traditional toys does not vary much with age. Non-digital objects and artefacts are highest at two (38.69%) and three (35.25%), while at the age of five, they reduce to 25.39%.

**Figure 6:**
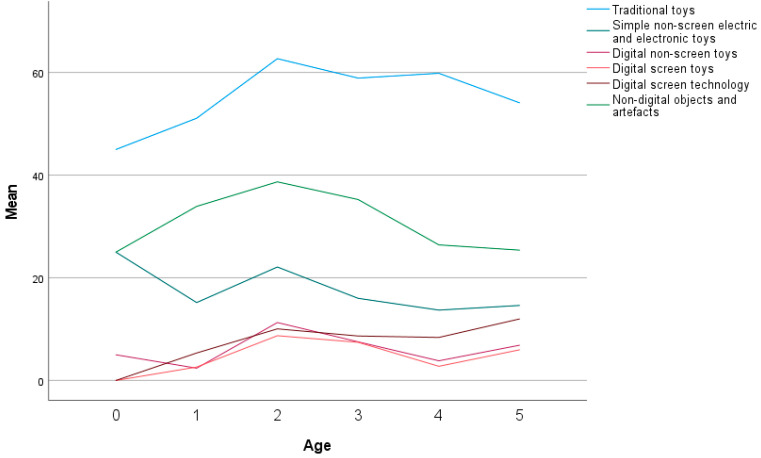
Parents’ estimation of playtime with traditional and digital toys according to child’s age.

With age (Figure 6), the amount of simple non-screen electric and electronic toys are decreasing, being highest at the age of two (22.10%). One interesting fact is observed at the age of two, indicating digital technology and toys have the largest share among all age groups, digital non-screen toys (11.28%), digital screen technology (10.05%), and digital screen toys (8.72%). The age of three is followed by also significant share of digital non-screen toys (7.52%), digital screen technology (7.43%), and digital screen toys (8.66%). Digital technology and toys increase at the age of two and then decrease towards the age of four before increasing again at the age of five.

At age two, all toy categories are growing: traditional, artefacts, simple electric, non-screen digital, digital screen toys and digital screen technology.

**Figure 7:**
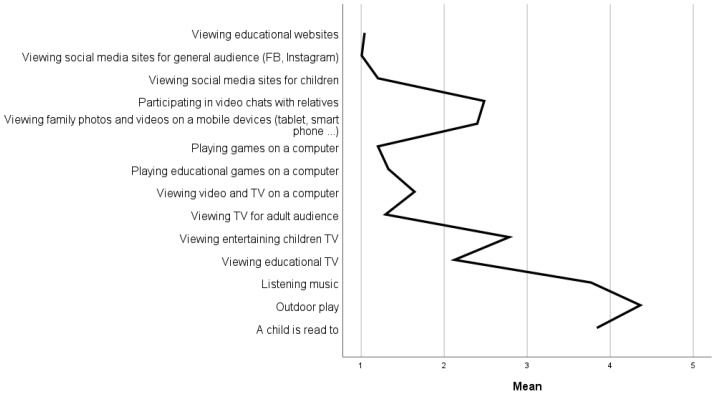
Parents’ estimation of child’s daily playtime activity.

Parents’ estimation of their child’s daily playtime activity is presented in Figure 7. The highest mean was for outdoor play (M = 4.36, SD = 0.61), which was also high in all age groups. The second was reading to a child (M = 3.84, SD = 0.88) and also very high in all age groups, with the highest at the age of one (M = 4.12, SD = 0.70). Following was listening to music (M = 3.77, SD = 0.88), high in all age groups and the highest in the age of one (M = 4.18, SD = 0.90). All other age categories had lower means.

Looking at screen activities, the highest mean was in participating in video chats with relatives (M = 2.48, SD = 0.83) and viewing family photos and videos (M = 2.40, SD = 0.82).

Participating in video chats with relatives, the highest mean was for four-year-olds (M = 2.78, SD = 0.79) and two-year-olds (M = 2.60, SD = 0.91). Again, viewing photos and videos with relatives is the highest mean for two-year-olds (M = 2.68, SD = 1.03) and four-year-olds (M = 2.59, SD = 0.80).

**Figure 8:**
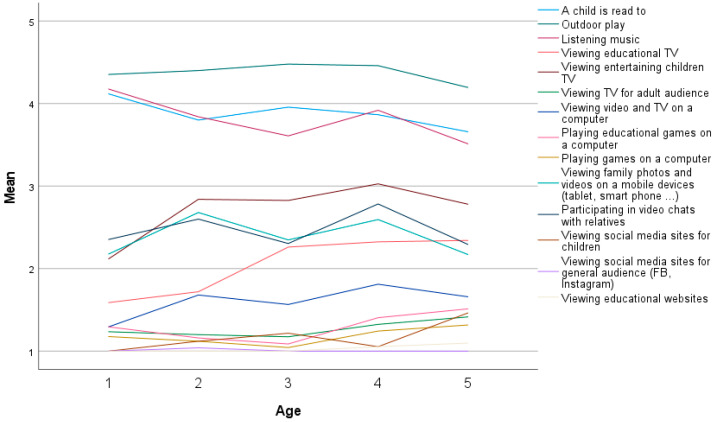
Parents’ estimation of child’s daily playtime activity according to child’s age.

Referring to age groups (Figure 8) regarding TV viewing, the first was viewing entertaining children’s TV (M = 2.78, SD = 1.04), and the highest was for four-year-olds (M = 3.03 SD = 0.93). After this was viewing educational TV (M = 2.13, SD = 0.90) with the highest mean for five-year-olds (M = 2.34, SD = 0.85).

Looking at the gender differences, there is a higher mean for girls in being read to (M = 3.97, SD = 0.83) vs. boys’ mean (M = 3.72, SD = 0.90). The outdoor play had a higher mean for boys (M = 4.43, SD = 0.52) vs. girls’ mean (M = 4.28, SD = 0.69). Girls had a lover mean for listening to music (M = 3.85, SD = 0.92) vs. boys’ mean (M = 4.70, SD = 0.83). TV viewing indicated a higher mean for educational TV for boys (M = 2.16, SD = 0.92) vs. girls’ mean (M = 2.09, SD = 0.88). In viewing entertaining TV, girls had a higher mean (M = 2.81, SD = 1.02) vs. boys’ mean (M = 2.76, SD = 1.07).

Spearman’s correlational coefficient indicates a correlation on a level *p* = 0.01 level (two-tailed) between age and viewing educational TV (ρ = 0.309, *p* < 0.001), playing educational games on the computer (ρ = 0.333, < 0.001), playing games on the computer (ρ = 0.247, *p* < 0.001), using educational contents on a computer (ρ = 0.317, *p* < 0.001), viewing social media sites for children (ρ = 0.224, *p* < 0.001). A correlation on a level *p* = 0.01 (two-tailed) was indicated between age and listening to music (ρ = −0.188, *p* < 0.024), viewing TV for an adult audience (ρ = 0.192, *p* < 0.022), viewing educational websites (ρ = 0.198, *p* < 0.018).

Spearman’s correlational coefficient indicates a correlation on a level *p* = 0.01 level (two-tailed) between the use of digital screen technology and some daily child activities (Table 3). The highest correlation is between playing games on a mobile device (ρ = 0.457, *p* < 0.001) and a computer (ρ = 0.421, *p* < 0.022). Following are viewing video and TV (ρ = 0.391, *p* < 0.001), educational games (ρ = 0.326, *p* < 0.001) and educational contents (ρ = 0.313, *p* < 0.001). The negative correlation is identified with reading to a child (ρ = −0.391, *p* < 0.001). Small correlations can be seen in Table 3.

**Figure 9:**
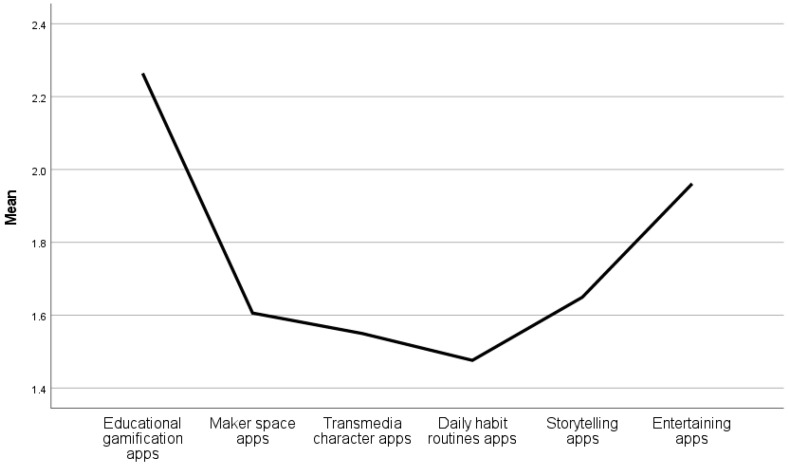
Application types that parents offer to a child.

**Figure 10:**
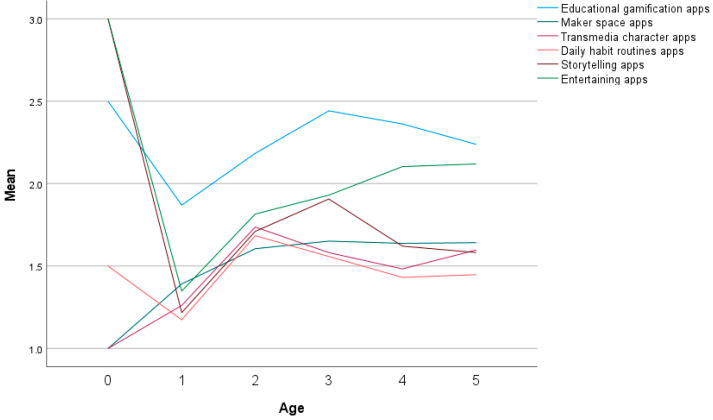
Application types that parents offer to a child according to the child’s age.

Application types that parents offer to a child are presented in Figure 9. The highest mean is in offering educational apps to children (M = 2.26, SD = 1.16). According to age group (Figure 10), the highest mean is for three-year-olds M = 2.44, SD = 1.29)

Gender mean differences are small with larger how much parents offer entertaining apps to a child, with the girls’ showing lower means (M = 1.86, SD = 1.12) vs. boys’ mean (M = 2.05, SD = 1.03).

The correlations of the use of digital screen technology with applications offered by parents to a child are presented in Table 4. The use of digital screen technology indicates correlation with the application offered to a child and among them on a level *p* = 0.01 level (two-tailed) with entertaining apps (ρ = 0.475, *p* < 0.001), transmedia character apps (ρ = 0.379, *p* < 0.001), and daily habit routines apps (ρ = 0.361, *p* < 0.001).

The use of digital screen technology indicates correlation with the application offered to a child on a level *p* = 0.01 level (two-tailed), and among them, the highest is with entertaining apps (ρ = 0.475, *p* < 0.001), transmedia character apps (ρ = 0.379, *p* < 0.001), and daily habit routines apps (ρ = 0.361, *p* < 0.001).

## 4. Discussion and Conclusions

Kindergarten curriculum is under the societal and economic pressure of a digitalized society and developing 21st-century skills and must address recommendations of public health agencies advocating for caution in technology use. Health organizations warn against the risks to health in a child’s psychophysical and emotional development [24,25,26,27]. Early learning researchers recognize the realities of children’s home environments and the growing role of digital technology in preschoolers’ lives [10,11]. Sociocultural theories originated in Vygotsky [12], highlighting the role of a child’s cultural environment and the communication and interaction of a child in the primary home and secondary kindergarten environment. Digital technology has been recognized as an important social and learning tool that influences social practices [13]. In these dynamic contexts of antagonizing views “pro et contra”, reported parents’ concerns about digital technology use in kindergarten [23], which was the focus of the study.

Slovene kindergarten curriculum reform took place in the last decade of the 20th century when digital social and learning tools had been recognized for early literacy [3,4,5], developing mathematical concepts [6], and learning about ICT and manipulating it [7]. Decades after reform, Edwards [52] reports that in some countries, digital technology is not integrated into the kindergarten curriculum and is necessary to bridge the gap between the consumption of entertaining popular culture and learning technology.

Slovene curriculum published in 1999 [1] covers important areas according to Plowman et al. [7], and among them, learning about digital technology and how to manipulate it, expanding knowledge about the world in curricular areas and developing affective, social and cognitive dispositions for learning, which strengthened by guidelines published in 2016 [20]. The Slovene play-based learning kindergarten curriculum originated from sociocultural theory and recognized the child’s primary cultural environment with cultural artefacts, which are among the main principles of kindergarten education in partnership with parents and interaction between primary (home) and secondary (kindergarten) environments.

As a child’s practices in the primary environment should inform kindergarten teachers, this study examines how parents, which formed a non-randomized sample of 306 participants, construct preschoolers’ home environment and play in terms of a share of digital play vs. traditional. Examined was how they perceive digital technology and digital play and its role in a child’s development and learning vs. traditional play. Educators informed with child’s digital practices at home could make informed decisions integrating learning technology and support parents when they have concerns regarding digital technology.

Conflicting views between economics and recommendations advocating caution antagonize parents and require educators’ evidence-based interventions. The findings of this study among Slovene parents indicate that digital technology can create conflict among parents. They are strongly aware of risks and potential harms that health organizations address and, on the other hand, recognize the benefits of technology for a child’s learning. The item with the highest mean was I want the child to enjoy their childhood and not become addicted to technology (M = 4.48, SD = 0.73). High means are also for items: the use of a computer can promote long-term physical, emotional, or intellectual developmental damage (M = 4.15, SD = 0.90), and my child should first learn to interact with the physical world; they have a lifetime to interact with the virtual world (M = 4.12, SD = 0.99). Parents would limit screen time to an hour daily (M = 4.06, SD = 0.99), and they believe that screen technology could only be used under the supervision of parents (M = 4.07, SD = 0.85). Other parents indicated concern about digital technology’s influence on a child’s development and learning (M = 3.79, SD = 1.01) and believe that a child develops better without digital technology (M = 3.69, SD = 1.06). Parents also believe that a child will not fall behind other children academically if his or her use of technology tools is restricted in the early years (0–3 years) (M = 3.70, SD = 1.37).

Parents hold a belief that introducing technological tools at a young age prepares children better for tomorrow’s workforce (ρ = 0.476, *p* < 0.001) and in early childhood, children can use technology as a learning tool (ρ = 0.359, *p* < 0.001).

The study identified correlations between digital screen technology use among children with parents’ attitudes toward digital technology in early childhood. The Spearman correlation coefficient indicated negative correlations of digital screen technology used by a child, with items indicating parents’ concerns about digital technology. The highest correlations are for items: the use of a computer can promote long-term physical, emotional, or intellectual developmental damage (ρ = −0.406, *p* < 0.001), a child develops better without technology (ρ = −0.436, *p* < 0.001), I want the child to enjoy their childhood and not become addicted to technology (ρ = −0.421, *p* < 0.001), and I am concerned about the effect technology can have on a young child’s brain development and learning (ρ = −0.384, *p* < 0.001). In the study in the US, parents expressed positive attitudes towards media at an early age to be vital for a child’s development, showing disagreement with the limitations of screen time, according to [31].

Parents who offer digital screen technology to their child agree (identified positive correlations) with items media exposure at a young age (0–3 years) is important for early brain development (ρ = 0.451, *p* < 0.001), introducing technological tools at a young age prepares children better for tomorrow’s workforce (ρ = 0.476, *p* < 0.001) and in early childhood, children can use technology as a learning tool (ρ = 0.359, *p* < 0.001). Among children’s daily activities, worryingly, the correlations in our sample show that technology use negatively correlates with reading.

Looking at the share of toys in playtime in the post-digital era, as indicated in our sample, it is obvious that digital technology and toys hold a share of about a fourth of all playtime (21.42%). Digital technology among this is the largest share (9.38%), digital non-screen toys (6.52%), and digital screen toys (5.52%). The largest share still belongs to traditional toy play (57.50%), followed by non-digital objects and artefacts (30.57%) and in third place are simple non-screen electric and electronic toys (16.05%).

The use of digital screen technology indicates a very high correlation with the entertaining applications offered to a child (ρ = 0.475, *p* < 0.001), followed by transmedia character apps (ρ = 0.379, *p* < 0.001), and daily habit routines apps (ρ = 0.361, *p* < 0.001). Recent research shows that digital apps for early learning may not be of such high quality [46]. When buying digital technology and applications, parents are facing a dilemma of whether they allow their child to use digital technology in order not to prevent the child from developing the skills and competencies necessary for education and the labor market, even at the expense of uncertainty regarding the achievement of the promised educational results and hazard for the child’s health and development. The purchase of modern technological devices is also a significant economic burden for parents, for which it is difficult for them to judge the effectiveness and sense of the purchase. When choosing technology and applications, it is difficult for parents to decide whether apps have educational functions.

Findings in the Slovene sample under examination indicated parents’ diversified perceptions on one side benefits for child’s skills and acknowledgements of risks. In the post-digital era, early learning is facing societal contexts of the complex state of digital technology introducing diverse effects for early learning and development, the positive and enabling potential and negative causing the condition or an object of hazard or risk. Informed use requires awareness of its influences on learning and development in terms of changing cognition, socioemotional and psycho-physical processes. Learning benefits reported at the end of the 20th century’s digitalized society were in all curricular areas [3,4,5,6]. During the last decade of the 21st century in the post-digital era, the highlight is emergent literacy involving digital literacy with multimodal texts [21]. Its cultural tool is digital storytelling as an authentic form of expression [28].

A child’s early literacy development occurs in the primary environment and reflects the parent’s skills and competencies [21]. As the new literacy is defined online and offline and with multimodal text, such practices should take place in the child’s primary environment, home. The accessibility to learning technology, therefore, could only be enabled by a competent caregiver, parent, or educator who is informed by new learning technology and applications which offer satisfactory educational functions. Accessibility is a matter of competency and not necessarily of technology accessibility. Therefore, the kindergarten principle of equity involves educators’ competency in digital learning technology. When parents are not informed by learning technology engaging digital multimodal text production, the kindergarten should have a leading and not just compensatory function.

Regarding potential negative effects, indicated excessive use, which could have potentially harmful effects, including impaired emotional and social intelligence, heightened attention-deficit symptoms, technology addiction, social isolation, impaired brain development, and disrupted sleep [60] (p. 1). Research indicates sustained engagement in physical activity and an increasing number of young children have insufficiently developed locomotor skills [61]. Health and physical activity habits, which could cause preschool children’s motor delays [61], could have been connected with a child’s engagement with digital technology when they could have outdoor play and physical activity.

Therefore, integrating digital applications for skills training and healthy digital habits and routines is also needed in early learning. The lack of informed use presents risk and places new demands on educators and parents for a child’s healthy development.

The reform of the Slovene kindergarten curriculum at the end of the 20th century highlighted the kindergarten’s compensatory role in a child’s emergent literacy. According to the digitalization of society, introduced were media production and reflection enable technology to learn (1) about digital technology and how to manipulate it, (2) expanding knowledge about the world in curricular areas, and (3) developing affective, social and cognitive dispositions for learning.

Kindergarten curriculum is meeting complex post-digital society needs with societal practices seamlessly integrating digital technology and constantly evolving digital technology innovation. This also brings new risks and hazards. Curricular objectives need to integrate digital learning technology (1) in literacy development involving multimodal texts (2) subject-specific in computer technology and the digital world enabling computational thinking and skills, (3) digital technology transforming natural science, social science, and arts and (4) and daily practices and habits involving digital practices for healthy life and well-being of a child. The curriculum needs to inform all stakeholders from professional areas, including psychology and health.

The curriculum’s objectives with activities recommendations need to be transparent for all curricular areas. The special transversal area is using digital learning technology to develop affective, social and cognitive dispositions for learning.

The study’s limitations come from the non-randomized sample, making it impossible to determine anything more than just considering and interpreting the sample. However, this exploratory study allows us to consider and prepare for future randomized studies to support preschoolers’ primary play environments and kindergarten classrooms. An educator’s role is increasingly involved in modelling learning technology for evidence-based early learning. Expected is the growth of research evidence and information from all important stakeholders, economic, health professionals, and educators. To navigate these information expansions, parents will need constant guidance and interaction with educators. The educators’ role modelling in introducing learning technology in early learning could importantly contribute to a child’s health and well-being. Future studies must address educators’ professional knowledge of the contributions of digital technology and its support of affective, social, and cognitive dispositions for learning.

## Figures and Tables

**Table 1:** Spearman’s correlations between the uses of digital screen technology with parents’ attitudes—the significant correlations are presented.

Item	Item	Correlation Coefficient	Sig. (2-Tailed)
The use of digital screen technology	Media exposure at a young age (0–3 years) is important for early brain development.	0.451 **	<0.001
	A child will not fall behind other children academically if his or her use of technology tools is restricted in the early years (0–3 years).	−0.290 **	<0.001
	Children under the age of 2 years should have no TV screen time.	−0.300 **	<0.001
	Children between 2 and 5 should limit their screen technology to 1 h a day.	−0.284 **	<0.001
	Children between 2 and 5 should only use screen technology with their parents present.	−0.384 **	<0.001
	The use of a computer can promote long-term physical, emotional, or intellectual developmental damage.	−0.460 **	<0.001
	Introducing technological tools at a young age prepares children better for tomorrow’s workforce.	0.476 **	<0.001
	In early childhood, children can use technology as a learning tool.	0.359 **	<0.001
	Young children develop better without technology.	−0.436 **	<0.001
	My child should first learn to interact with the physical world; they have a lifetime to interact with the virtual world.	−0.463 **	<0.001
	I am concerned about how technology can affect a young child’s brain development and learning.	−0.384 **	<0.001
	I want the child to enjoy their childhood and not become addicted to technology.	−0.421 **	<0.001
	Due to the intensive use of digital technology in the family, parents are reading less and less to their children.	−0.168	0.025

** *p* < 0.001 level.

**Table 2:** Spearman’s correlations between the uses of digital screen technology with parents’ perceptions of toys –significant correlations are presented.

Item	Item	Correlation Coefficient	Sig. (2-Tailed)
The use of digital screen technology	During the play, a child intensively communicates with the person/s present.	0.292 **	<0.001
	During playtime, the most important communication is between a child and the engaged person.	–0.152 *	0.033
	Children under age three should not use technological toys.	–0.284 **	<0.001
	Technological toys enhance a child to be passive.	–0.203 **	0.004
	Some technological toys enhance a child to develop early literacy.	0.243 **	<0.001
	Technological toys are better than traditional ones.	0.405 **	<0.001
	With the help of technological toys, parents easily guide their child’s play.	0.220 **	0.002
	Preschoolers better develop without technology.	–0.364 **	<0.001
	Technological toys please children more than traditional	0.153 *	0.032

** *p* < 0.001 level. * *p* < 0.05 level.

**Table 3:** Spearman’s correlations between the uses of digital screen technology with child’s daily activity; the significant correlations are presented.

Item	Item	Correlation Coefficient	Sig. (2-Tailed)
The use of digital screen technology	A child is read to	−0.391 **	<0.001
	Viewing educational TV	0.176 *	0.035
	Viewing entertaining children’s TV	0.190 *	0.023
	Viewing video and TV on a computer	0.223 **	0.007
	Playing educational games on a computer	0.326 **	<0.001
	Playing games on a computer	0.421 **	<0.001
	Using educational content on a computer	0.313 **	<0.001
	Playing educational games on mobile devices	0.210 *	0.012
	Viewing video and TV on mobile devices (tablet, smartphone…)	0.391 **	<0.001
	Playing games on mobile devices (tablet, smartphone…)	0.457 **	<0.001
	Using apps on mobile devices (tablet, smartphone…)	0.386 **	<0.001
	Viewing social media sites for children	0.224 **	0.007
	Digital screen toys	0.244 *	0.018

** *p* < 0.001 level. * *p* < 0.05 level.

**Table 4:** Spearman’s correlations of the use of digital screen technology with applications offered by parents; significant correlations are presented.

Item	Item	Correlation Coefficient	Sig. (2-Tailed)
The use of digital screen technology	Transmedia character apps	0.379 **	<0.001
	Daily habit routines apps	0.361 **	<0.001
	Entertaining apps	0.475 **	<0.001

** *p* < 0.001 level.

## Data Availability

Not applicable. The data of the present study is not openly available, as the participants in this study did not agree to their data being shared publicly during the data collection phase of the study.
